# Effectiveness and Acceptance of Technology-Based Psychological Interventions for the Acute Treatment of Unipolar Depression: Systematic Review and Meta-analysis

**DOI:** 10.2196/24584

**Published:** 2021-06-13

**Authors:** Moritz Köhnen, Levente Kriston, Martin Härter, Harald Baumeister, Sarah Liebherz

**Affiliations:** 1 Department of Medical Psychology University Medical Center Hamburg–Eppendorf Hamburg Germany; 2 Department for Clinical Psychology and Psychotherapy University of Ulm Ulm Germany

**Keywords:** internet, digital health, digital mental health, telephone, psychotherapy, depressive disorder, systematic review, meta-analysis, technology-based psychological interventions

## Abstract

**Background:**

Evidence on technology-based psychological interventions (TBIs) for the acute treatment of depression is rapidly growing. Despite extensive research in this field, there is a lack of research determining effectiveness and acceptance of TBIs considering different application formats in people with a formally diagnosed depressive disorder.

**Objective:**

The goal of the review was to investigate the effectiveness and acceptance of TBIs in people with diagnosed depression with particular focus on application formats (stand-alone interventions, blended treatments, collaborative and/or stepped care interventions).

**Methods:**

Studies investigating adults with diagnosed unipolar depressive disorders receiving any kind of psychotherapeutic treatment delivered (at least partly) by a technical medium and conducted as randomized controlled trials (RCTs) were eligible for inclusion. We searched CENTRAL (Cochrane Central Register of Controlled Trials; August 2020), MEDLINE, PsycINFO, PSYNDEX, CINAHL (January 2018), clinical trial registers, and sources of grey literature (January 2019). Two independent authors decided about study inclusion and extracted data. We performed random effects meta-analyses to synthesize the data.

**Results:**

Database searches resulted in 15,546 records of which 78 completed studies were included. TBIs delivered as stand-alone interventions showed positive effects on posttreatment depression severity when compared to treatment as usual (SMD –0.44, 95% CI –0.73 to –0.15, k=10; *I*²=86%), attention placebo (SMD –0.51, 95% CI –0.73 to –0.30; k=12; *I*²=66%), and waitlist controls (SMD –1.01, 95% CI –1.23 to –0.79; k=19; *I*²=73%). Superior long-term effects on depression severity were shown when TBIs were compared to treatment as usual (SMD –0.24, 95% CI –0.41 to –0.07; k=6; *I*²=48%) attention placebo (SMD –0.23, 95% CI –0.40 to –0.07; k=7; *I*²=21%) and waitlist controls (SMD –0.74, 95% CI –1.31 to –0.18; k=3; *I*²=79%). TBIs delivered as blended treatments (providing a TBI as an add-on to face-to-face treatment) yielded beneficial effects on posttreatment depression severity (SMD –0.27, 95% CI –0.48 to –0.05; k=8; *I*²=53%) compared to face-to-face treatments only. Additionally, TBIs delivered within collaborative care trials were more effective in reducing posttreatment (SMD –0.20, 95% CI –0.36 to –0.04; k=2; *I*²=0%) and long-term (SMD –0.23, 95% CI –0.39 to –0.07; k=2; *I*²=0%) depression severity than usual care. Dropout rates did not differ between the intervention and control groups in any comparison (all *P*≥.09).

**Conclusions:**

We found that TBIs are effective not only when delivered as stand-alone interventions but also when they are delivered as blended treatments or in collaborative care trials for people with diagnosed depression. Our results may be useful to inform routine care, since we focused specifically on different application formats, formally diagnosed patients, and the long-term effectiveness of TBIs.

**Trial Registration:**

PROSPERO International Prospective Register of Systematic Reviews CRD42016050413; https://www.crd.york.ac.uk/prospero/display_record.php?ID=CRD42016050413

**International Registered Report Identifier (IRRID):**

RR2-10.1136/bmjopen-2018-028042

## Introduction

Depression is a common [1] and debilitating mental disorder for affected individuals (eg, experiencing difficulties in everyday life) [2] and society (eg, burden of disease caused by depression) [3]. There are many effective treatment options, especially psychotherapeutic and pharmacological treatments, for people diagnosed with unipolar depression [1,4]. Despite the high prevalence, burden, and presence of many effective treatment options, depression is still undertreated [5].

Technology-based psychological interventions (TBIs) are seen as promising tools to supplement mental health care [6]. TBIs comprise a heterogeneous group of interventions [7] that can be delivered in different clinical phases of depression management (eg, acute treatment, relapse prevention); within these phases, they can be distinguished concerning their application format: stand-alone interventions, blended treatments, collaborative and/or stepped care interventions. In line with the German guideline for unipolar depression [1], we defined acute treatment as the treatment of an acute/present unipolar depressive episode aiming to reduce symptom burden so that response or remission of patients may be achieved. This clinical phase is differentiated from continuation and maintenance treatment and relapse prevention, which aim to further stabilize (responded or remitted patients of the acute treatment) and prevent relapse (or recurrence of new episodes) in the long term among people being at high risk. Additionally, TBIs vary in technical aspects (eg, delivery via videoconferencing tools), amount of human support, and theoretical background of the intervention [7]. Due to considerable diversity among TBIs and extensive research efforts capturing effectiveness and acceptance of TBIs for the acute treatment phase [8-10], there is need to address important neglected issues concerning TBIs.

First, TBIs in depression have already been widely researched resulting in high-quality evidence [11], and certain moderators influencing the success of treatment have been identified (eg, guided TBIs result in lower dropout rates than unguided TBIs) [8]. However, guideline recommendations are still limited to the general effectiveness of specific TBIs (eg, computerized cognitive behavioral therapy [cCBT] [1,4]). Additionally, there is no systematic review examining the effectiveness and acceptance of TBIs in the acute treatment phase regarding different application formats, even though the evidence base is available [11]. TBIs can be delivered as stand-alone interventions (TBIs replacing face-to-face [f2f] treatment), as blended treatments (combining TBIs and f2f treatment), or as part of stepped (eg, TBIs are used as a low-threshold initial treatment option for people with mild-to-moderate depressive disorder) and/or collaborative care models (TBIs may be provided alongside different treatment components, such as a TBI offered in addition to a care manager and general practitioners’ care; see section *Application Formats of TBIs* for details). Blended treatments are usually conducted within a superiority (providing a full TBI alongside a full f2f treatment) or noninferiority (replacing some elements of f2f treatment by providing a TBI instead) trial design addressing different research questions (dose-response research focus vs cost-utility focus). A recent initiative considering both patients and clinicians emphasized top 10 research priorities in digital mental health [12]. One priority was to determine how treatment outcomes can be maximized by combining treatment options (eg, psychotherapy) with digital mental health interventions (ie, blended treatments). Considering application formats is of interest from the perspective of patients and clinicians, as it may help to determine effectiveness and acceptance of TBIs in a more differentiated manner, which may be relevant to inform clinical practice.

Second, the vast majority of research syntheses in this field included mixed populations based on symptom severity cutoff scores or the presence of diagnoses, providing valuable information on the effectiveness of interventions. To the best of our knowledge, there is only one systematic review evaluating internet- and mobile-based interventions in people with formally diagnosed depression; however, it is limited to waitlist control group comparisons [13]. In light of a comprehensive evidence base for TBIs in acute treatment [11] and the necessity of diagnoses to initiate treatment in mental health care, we focused only on studies requiring diagnosis of depression with the aim of determining the effectiveness and acceptance of TBIs. Additionally, high-quality evidence (RCTs) in clinical samples with diagnosed depression is the preferred source of evidence for the development and updating of clinical treatment guidelines such as the German [1] and United Kingdom [4] guidelines for depression.

Finally, to date there is no clarity regarding whether treatment effects achieved by TBIs are stable over time, since most reviews have focused on posttreatment intervention effects and have not considered long-term outcome data (for example, Karyotaki et al [14]).

By focusing specifically on different application formats, on people diagnosed with depression, and on long-term effectiveness of TBIs, we hope to provide a comprehensive evidence base that may be more useful to inform routine care than already existing evidence syntheses.

In summary, our main aim is to investigate posttreatment and long-term effectiveness and acceptance of TBIs delivered to people with diagnosed depression in the acute treatment phase, addressing the following research questions:

1. How effective and acceptable are TBIs delivered as stand-alone interventions compared to f2f treatment, attention placebo, treatment as usual (TAU), waitlist and no-treatment controls, and other TBIs?
2. How effective and acceptable are TBIs delivered as blended treatments (TBI plus f2f treatment) compared to f2f treatment (including psychotherapy, medication, TAU)?
3. How effective and acceptable are TBIs delivered as stepped and/or collaborative care approaches compared to TAU?

## Methods

The study was part of a larger research synthesis project (comparative effectiveness of Technology-Based Interventions in Different Steps of Depression Care [TIDECA]) that was prospectively registered with International Prospective Register of Systematic Reviews (PROSPERO) [CRD42016050413] and described in the study protocol published elsewhere [15].

### Search Strategy

The search was not limited by date, language, or publication status. We contacted first authors of all included publications for additional information on further (un)published trials and specific study information (see Köhnen et al [15] for details on the literature search/strategy).

### Selection Criteria

See study protocol [15] for more details on eligibility criteria. Our inclusion criteria were (1) at least 80% of sample having a diagnosed unipolar depression (assessed by criteria of a formal classification system or by conducting a diagnostic interview [eg, F32.x, F33.x, or F34.1 according to the *International Statistical Classification of Diseases and Related Health Problems, 10th Revision*]) with any comorbidities in the acute treatment phase for depression and consisted of adults aged 18 years and older, (2) intervention was at least partly delivered through technical devices (eg, telephone, smartphone, computer), (3) intervention was based on an explicit psychotherapeutic theory, and (4) study was an individual or cluster RCT.

Our exclusion criteria were (1) participants were solely diagnosed by applying cutoff scores on symptom severity scales or when they had a depressive episode in the course of a bipolar disorder, (2) concurrent conditions (either somatic or mental) were the focus of the intervention, or (3) intervention provided solely psychoeducational content, patient decision aids, or depression management tools or focused exclusively on medication adherence.

### Application Formats of TBIs

**Figure 1:**
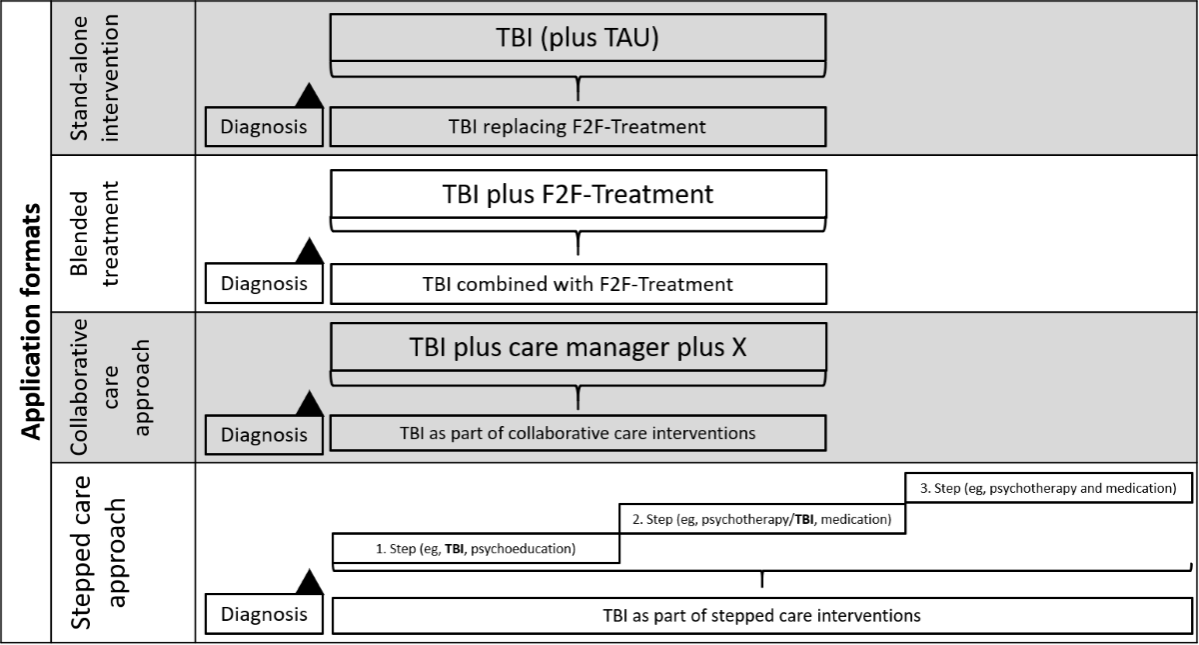
Illustration of potential application formats of technology-based psychological interventions.

Since we placed a special focus on application formats in this review, they are presented visually in Figure 1. We applied a rather broad definition for blended treatments, since we included all studies that provided any type of f2f treatment tailored to depression (eg, psychotherapy, medication, depression specific general practitioner care) in addition to TBIs irrespective of the study’s definition/label. In contrast, trials concurrently providing TAU in addition to TBIs were not considered blended treatments (but considered for the comparison TBI vs TAU) if TAU consisted of systematically offered generic treatments (eg, general practitioner care for all participants) that were not specifically tailored to depression. Since RCTs for blended treatment may be delivered in different designs (eg, superiority, noninferiority) resulting in content-related heterogeneity of interventions (eg, fewer therapeutic contacts), we decided to conduct meta-analyses separately.

### Selection Procedure

**Figure 2:**
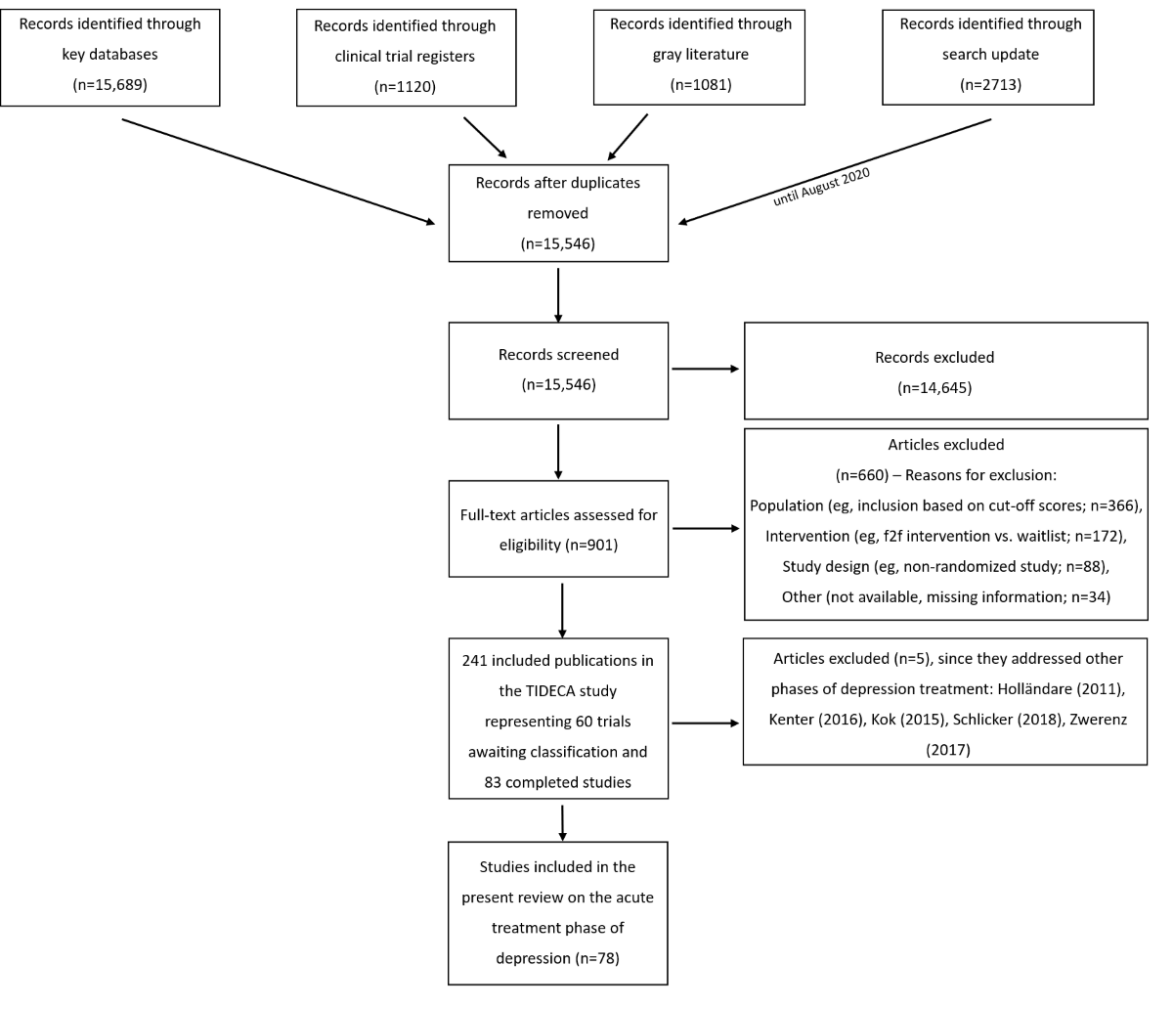
Preferred Reporting Items for Systematic Reviews and Meta-Analyses (PRISMA) flowchart.

The study flowchart is presented in Figure 2. Electronic searches yielded 20,603 records. After deduplication, 15,546 records were screened by title and abstract. Two reviewers (MK, SL) independently screened the first 100 records for inclusion. Since the interrater reliability for this sample was found to be high (98%), only one reviewer (MK) screened the remaining records in the course of the title/abstract screening. The second reviewer (SL) assessed publications labeled unclear by the first reviewer. Selected full-text articles (n=901) were subsequently assessed for inclusion by 2 independent reviewers (MK, MD). Discrepancies were resolved by discussion with a third reviewer (SL). In total, 241 publications representing 143 trials (83 completed studies and 60 ongoing studies awaiting further classification) fulfilled all inclusion criteria for the TIDECA study [11]. Of those, 78 completed studies assessed the acute treatment phase.

### Data Extraction

See Köhnen et al [15] for detailed information on extracted data and extraction procedure.

### Quality Appraisal

Risk of bias was independently assessed by 2 reviewers (from a group of 5 reviewers: MK, EW, MD, SL, TS) following Cochrane guidance (including the following domains for RCTs: random sequence generation, allocation concealment, blinding of participants and personnel, blinding of outcome assessment, incomplete outcome data, selective outcome reporting, and other bias) [16]. In line with a previous operationalization [17], we specified the domain *other bias* using the following 3 categories: insufficient treatment adherence, allegiance bias, and attention bias. Selective outcome reporting was categorized as *unclear risk* (trial registration or study protocol were missing or there was a deviation in one secondary outcome) or *high risk* (there were deviations in one primary or ≥2 secondary outcomes that could not be justified by the study authors). Disagreements were resolved by discussion or by consulting another reviewer (SL). Interrater reliability for risk of bias ratings was calculated to be 74%.

### Data Analysis

Meta-analyses were computed applying random effects models [18] since we assumed that heterogeneity regarding the sample, treatment, and methodological features of the included studies would be best captured by assuming that moderately diverging study-specific effect estimates are distributed around a grand mean [19]. Results were visually displayed as forest plots.

Continuous data (posttreatment and long-term depression severity) were analyzed as standardized mean differences (SMDs). Dichotomous data ([any] dropouts) were analyzed using the risk ratio (RR). We calculated 95% confidence intervals for all estimates. In addition, we computed 95% prediction intervals (PIs) for meta-analysis (when possible) capturing the range in which the effect of a new study (in a different setting) is expected; PIs can be very imprecise when only a few studies are considered [20].

Studies with multiple treatment groups were considered by combining data from interventional study arms (ie, pooling of means and standard deviations for continuous data and summing up sample sizes and people with events for binary data) when possible to avoid a unit-of-analysis error [16].

In cases of missing or unclear data, we contacted the corresponding authors. Intention-to-treat (ITT) analyses were used when reported by the included studies. When ITT data were not reported, we used the analysis defined as primary by the authors of the trial. Data on dichotomous outcomes were excluded from data analysis if there were no events in either study arm, since the direction and magnitude of a potential effect is not indicated [16].

We assessed statistical heterogeneity in the included studies by using a Cochran Q test and quantified it using the *I*² statistic [21]. As defined in the study protocol [15], we considered *I*² values of 50% or more as indicators of relevant statistical heterogeneity requiring further exploration. If indicated, we explored heterogeneity either quantitatively by means of a priori (see Köhnen et al [15]) and post hoc subgroup analyses (if the number of studies was sufficient [≥10]) or narratively (if only a few studies were available [<10]). We tested for possible reporting biases and small-study effects using visual examination of funnel plots (when useful). Possible control interventions and comparisons of interests were prespecified in our protocol [15] and used to structure our results section. All meta-analyses were computed by using Review Manager 5.4 (Cochrane Collaboration); descriptive data (eg, mean age of included participants) and PIs were calculated using Excel 2013 (Microsoft Corp).

## Results

A table summarizing all meta-analytic results can be found in Multimedia Appendix 1.

### Study Characteristics and Quality of Included Studies

**Figure 3:**
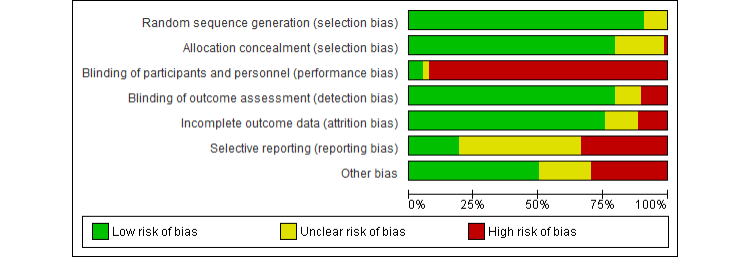
Risk of bias assessment across included studies (n=78).

Overall, the selected studies (n=78) included 13,180 participants ranging from 14 to 1089 per study. The mean age of participants was 45.15 (SD 12.01) years, and two-thirds (8029/11981, 67.01%) were female. TBIs in the included studies were delivered as stand-alone interventions (61/78; 78%), blended treatments (12/78; 15%), collaborative care (3/78; 4%), or stepped care trials (2/78; 3%). Duration of TBIs ranged from 1 week to 52 weeks, with most interventions lasting between 6 weeks and 12 weeks (median treatment length of 8 weeks). Interventions of 8 weeks’ duration were the most frequent (26/89; 29%) in the included studies (see Multimedia Appendix 2 [22-99] for baseline diagnoses). TBIs were based on 13 therapeutic rationales with most (83/101, 82.2%) based on CBT approaches (see Multimedia Appendix 3 for details). Concerning the applied technical medium, most TBIs were delivered via the internet (55/101, 54.5%), followed by telephone (12/101, 11.9%), offline computer programs (8/101, 7.9%), and videoconferencing tools (3/101, 3.0%). Additionally, 22.8% (23/101) of interventions applied more than one technical medium (internet-based treatment plus telephone support was most frequently [17/101, 16.8%] combined). The most common source of risk of bias was nonblinding of participants and personnel, selective reporting, and other bias (especially due to insufficient treatment adherence; Figure 3; see Multimedia Appendix 4 [22-99] for details).

### Stand-Alone Interventions

#### TBI Versus Face-to-Face Treatment

**Figure 4:**
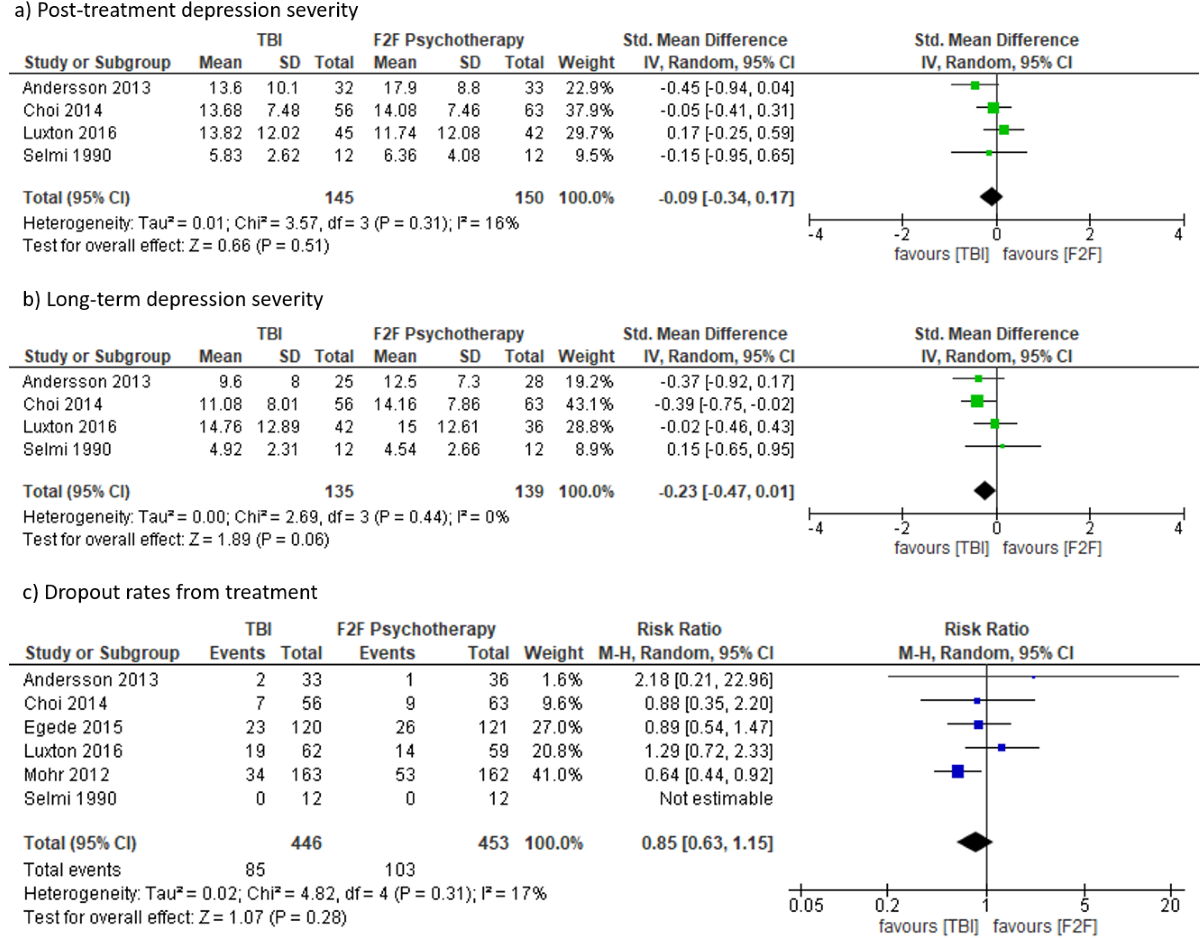
Forest plots on technology-based psychological intervention versus face-to-face-treatment.

There were 6 RCTs comparing TBIs with f2f treatments [23,32,36,58,66,84]; 4 delivered therapist-administered treatment via videoconferencing [32,36,58] or telephone [66], and 2 delivered guided internet-based [23] or computer-based treatment [84]. There was no significant difference in posttreatment (SMD –0.09, 95% CI –0.34 to 0.17; *I*²=16%; 95% PI –0.80 to 0.62) or long-term depression severity (2 months to 12 months; SMD –0.23, 95% CI –0.47 to 0.01; *I*²=0%; 95% PI –0.76 to 0.3) between TBI and f2f interventions. There was no statistically significant difference in dropout rates between interventions (RR 0.85, 95% CI 0.63 to 1.15; *I*²=17%; 95% PI 0.44 to 1.65; see Figure 4).

#### TBI Versus Treatment as Usual

**Figure 5:**
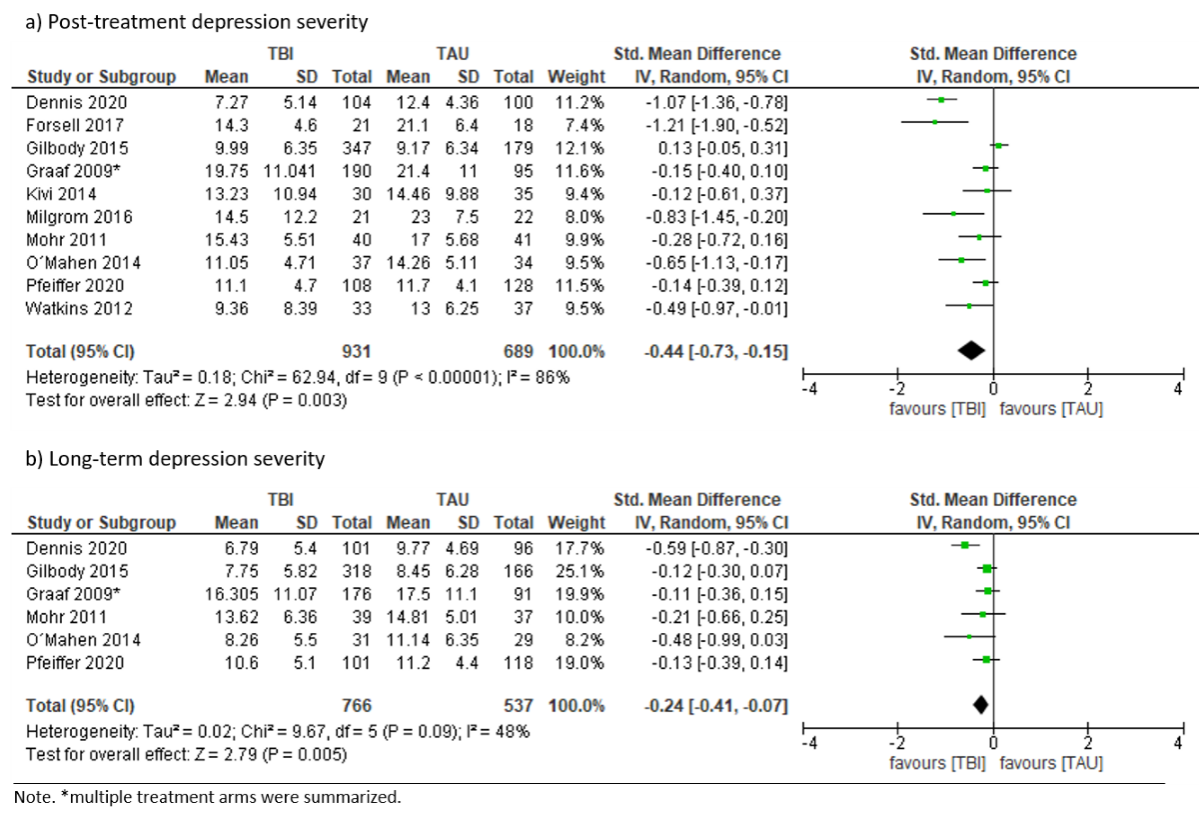
Forest plots on technology-based psychological intervention versus treatment as usual.

There were 12 RCTs testing TBIs against TAU [34,35,39-41,51,57,63,64,72,74,92], 8 of which explicitly stated that TAU was also administered in the TBI condition [34,35,39-41,57,74,92]. TBIs were delivered either with [39-41,51,63,72,74,92] or without [34,57] guidance or they were therapist-administered [35,64]. TAU consisted of care by a general practitioner [34,40,41,57,92], a heterogeneous mix of treatment options depending on resources and routines [51,63,72,74], care by community-based outpatient clinics and any non-Veterans Affairs facilities [64], and antenatal [39] or postpartum care [35]. Depression severity at posttreatment, with considerable heterogeneity (SMD –0.44, 95% CI –0.73 to –0.15; *I*²=86%; 95% PI –1.48 to 0.60, and in the long term (6 months to 12 months; SMD –0.24, 95% CI –0.41 to –0.07; *I*²=48%; 95% PI –0.70 to 0.22) was statistically significantly lower in the TBI condition (see Figure 5). Data on dropout rates were either not usable or missing. Prespecified subgroup analyses exploring heterogeneity for posttreatment depression severity were not conducted, as too few studies were available. Further exploration of heterogeneity did not reveal any specific source of variation. However, heterogeneity may be explained by the rather broad TAU condition, which consisted of various treatment options depending on the specific health care context where the intervention was delivered. Visual inspection of the funnel plot was not suspicious (Multimedia Appendix 5).

#### TBI Versus Attention Placebo

**Figure 6:**
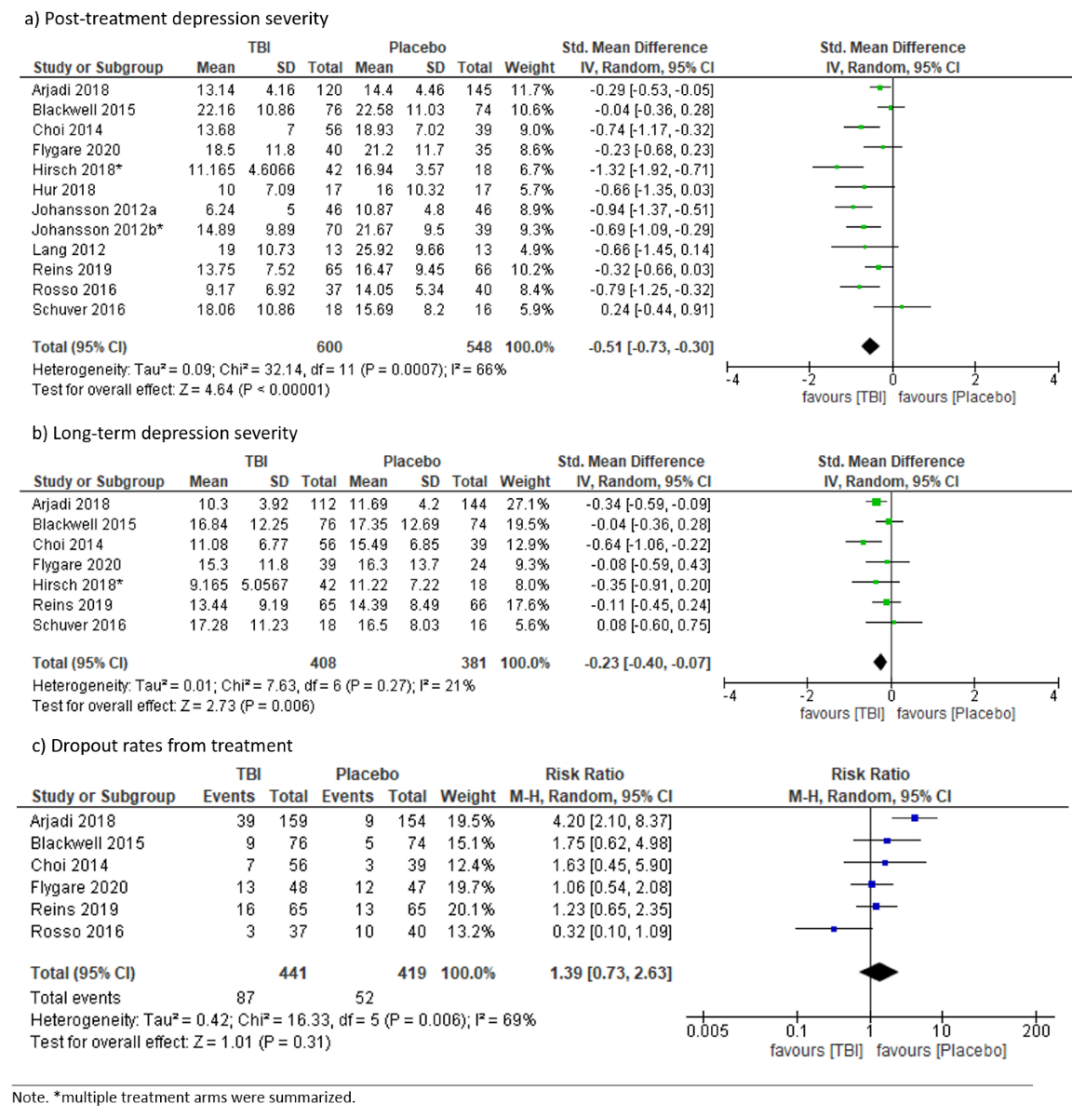
Forest plots on technology-based psychological intervention versus attention placebo.

Twelve RCTs tested TBIs against attention placebo controls, which consisted of online psychoeducation [24,37,48,76], participation in an online discussion forum [49], unspecific telephone support calls [32], neutral tasks [42], tasks without training contingency [27,54], symptom monitoring plus short check-in telephone calls [81], daily mood diary [44], and a walking and wellness control condition [83]. Depression severity was significantly lower at posttreatment in the TBI group than in the attention placebo group, with substantial heterogeneity (SMD –0.51, 95% CI –0.73 to –0.30; *I*²=66%; 95% PI –1.22 to 0.20). Follow-up depression severity was significantly lower in the TBI group (1 month to 12 months; SMD –0.23, 95% CI –0.40 to –0.07; *I*²=21%; 95% PI –0.56 to 0.10). Dropout rates did not differ statistically significantly between groups, with substantial heterogeneity (RR 1.39, 95% CI 0.73 to 2.63; *I*²=69; 95% PI 0.56 to 3.43; see Figure 6). Quantitatively exploring heterogeneity for posttreatment depression severity by using prespecified subgroups (technology of intervention delivery, amount of therapist guidance) was not conducted, as the study characteristics were strongly unevenly distributed. It may be possible that heterogeneity was driven by applying broad criteria for attention placebo controls resulting in a rather heterogeneous collection of control conditions. Heterogeneity for dropout rates may be explained by the largest study [24], which clearly favors the attention placebo condition (online psychoeducation) over the TBI condition resulting in low overlap with the other studies in regard to dropout rates. Removing this study from the analysis decreased heterogeneity (*I*²=23%) and did not alter the direction of the effect (RR 1.09, 95% CI 0.69 to 1.72). Visual inspection of the funnel plot (Multimedia Appendix 5) was not suspicious.

#### TBI Versus Waitlist Controls

**Figure 7:**
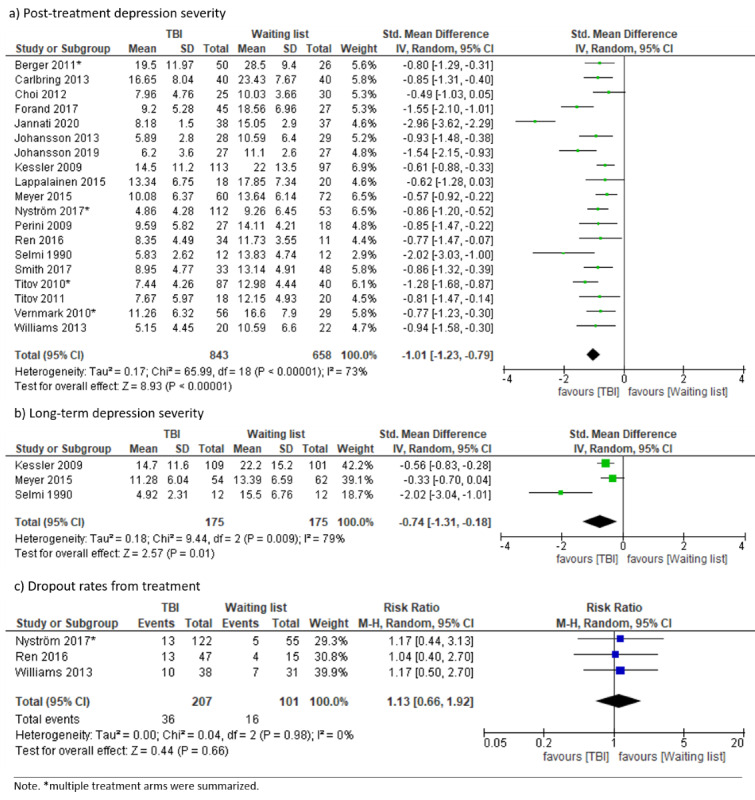
Forest plots on technology-based psychological intervention versus waitlist.

Twenty RCTs tested TBIs against waitlist controls. TBI arms of included studies applied guided [25,29,31,38,46,47,55,65,70,73,84,85,88,89,91], unguided [25,45,62,65,77,95], or therapist-administered [50,91] interventions. All but one study, which examined an offline computer program [84], used internet-based treatment. Depression severity was significantly lower at posttreatment in the TBI group compared to waitlist controls, with substantial heterogeneity (SMD –1.01, 95% CI –1.23 to –0.79; *I*²=73%; 95% PI –1.91 to –0.11). Follow-up depression severity was significantly lower in the TBI group, with considerable heterogeneity (2 months to 8 months; SMD –0.74, 95% CI –1.31 to –0.18; *I*²=79%; 95% PI –7.24 to 5.76). Dropout rates did not differ between groups (RR 1.13, 95% CI 0.66 to 1.92; *I*²=0%; 95% PI 0.04 to 35.12; see Figure 7). Heterogeneity for posttreatment depression severity (*I*²=73) may be explained by a potential outlying study [45], which was identified in the course of the search update yielding the largest effect in favor of TBIs (SMD –2.96, 95% CI –3.62 to –2.29) for this comparison. Excluding this study resulted in decreased heterogeneity (*I*²=41%) and did not alter the direction of the effect (SMD –0.89, 95% CI –1.04 to –0.74). Heterogeneity for long-term depression severity (*I*²=79) may be explained by an older study from 1990 [84], which had a shorter long-term time period (2 months) compared to the other studies (providing 6-month and 8-month long-term data [50,62]). Excluding this study resulted in decreased heterogeneity (*I*²=0%) and did not alter the direction of the effect (SMD –0.47, 95% CI –0.70 to –0.25). The funnel plot (Multimedia Appendix 5) was asymmetrical in the visual inspection.

#### TBI Versus No-Treatment Control

**Figure 8:**
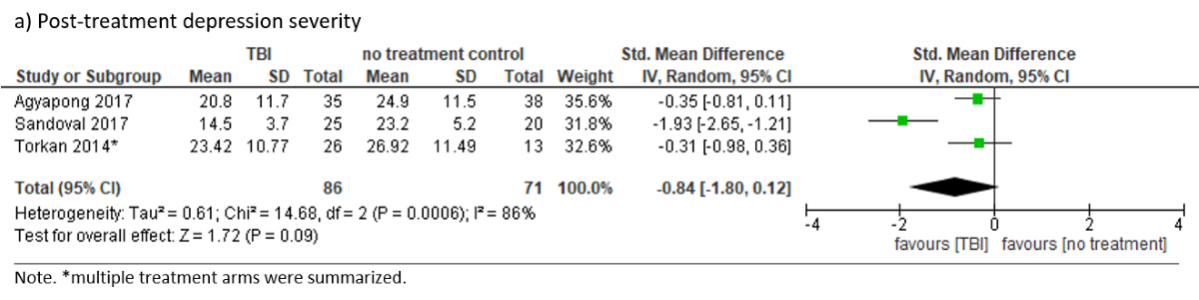
Forest plot for technology-based psychological intervention versus no-treatment control.

Three RCTs tested unguided TBIs against no-treatment controls [22,82,90], defined as a comparator where study participants did not receive any offer or encouragement for making use of immediate (eg, TAU) or delayed (eg, waitlist) treatment possibilities. There was no significant difference between TBIs and no-treatment controls at posttreatment (SMD –0.84, 95% CI –1.80 to 0.12; *I*²=86%; 95% PI –12.55 to 10.87; see Figure 8). Data on dropout rates were only available for one study [22], indicating that dropout rates did not statistically differ between conditions. Long-term data were not reported. Heterogeneity (*I*²=86) may be explained by an outlying, small-sample study with a large CI [82] favoring the TBI condition clearly, which might have been due to the provision of a more intensive TBI, as the TBI is either longer or needs a more active user engagement when compared to the other trials’ interventions. Excluding this study resulted in decreased heterogeneity (SMD –0.34, 95% CI –0.72 to 0.04; *I*²=0%) and did not change the direction of the effect.

#### Comparing Different Types of TBIs

Overall, 21 studies compared different TBIs competitively, 12 of which [25,34,40,42,49,65,68,70,80,88,90,91] compared multiple (2 or more) TBIs with a control group (eg, TAU). Thus, certain arms of these studies were suitable for other prespecified comparisons (eg, Gilbody et al [40] for TBI vs TAU). Nine of them compared TBIs versus another TBI [30,33,56,60,75,86,93,96,98] without having a further control group. For these studies, meta-analysis was not computed, since research foci of studies were too heterogeneous—they investigated different types of guidance (eg, telephone support vs email support) [56,75,98], treatment approaches [30,33,60,86,96], or delivery modes [93].

#### Other Comparisons

Two studies were identified during the search update that could not be matched to our comparisons [71,94]. One study compared a guided web-based CBT tool (iFightDepression) against an active control intervention receiving progressive muscle relaxation provided via a download link [71]. Another study investigated a TBI in combination with and without transcranial direct current stimulation [94].

### Blended Treatments

11 RCTs tested blended treatments against different f2f treatments. Six RCTs were identified combining TBIs with f2f psychotherapy versus f2f psychotherapy alone. In these trials, TBIs were delivered in addition to outpatient psychotherapy [26,52,97], inpatient psychotherapy [99], and psychotherapy treatment sessions where the setting was not specified [59,87]. Two RCTs were identified comparing a TBI in addition to medication versus medication alone [53,61], and 2 RCTs tested a TBI with f2f TAU against TAU [28,68]. Additionally, we identified one RCT [69] where blended treatment (f2f CBT and internet-based CBT) was provided alongside TAU (psychiatric treatment) compared to TAU. Overall, 8 superiority [26,28,53,61,68,69,97,99] and 3 noninferiority trials [52,59,87] applying blended treatments were identified.

#### Noninferiority Trials

**Figure 9:**
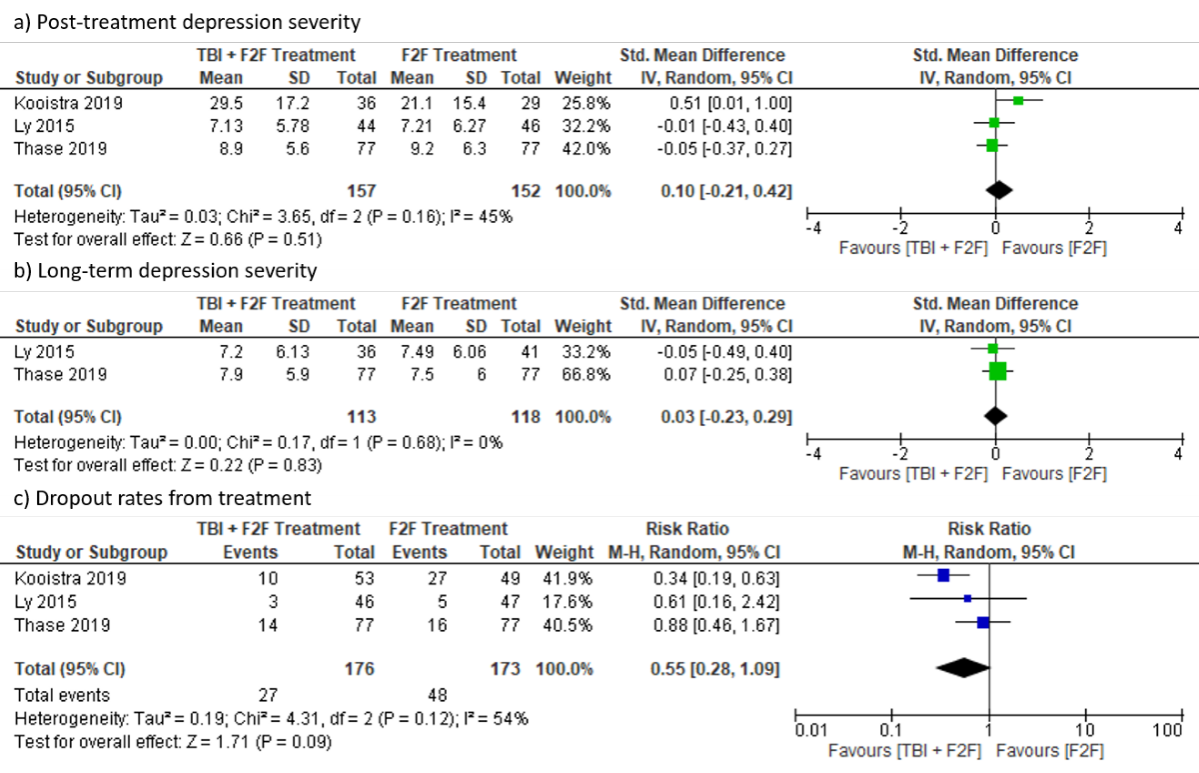
Forest plots for blended treatments (noninferiority trials).

There was no statistically significant difference between groups concerning posttreatment depression severity (SMD 0.10, 95% CI –0.21 to 0.42; *I*²=45%; 95% PI –2.91 to 3.12), long-term (6 months) depression severity (SMD 0.03, 95% CI –0.23 to 0.29; *I*²=0%), or dropouts (RR 0.55, 95% CI 0.28 to 1.09; *I*²=54%; 95% PI 0 to 663.21; see Figure 9).

#### Superiority Trials

**Figure 10:**
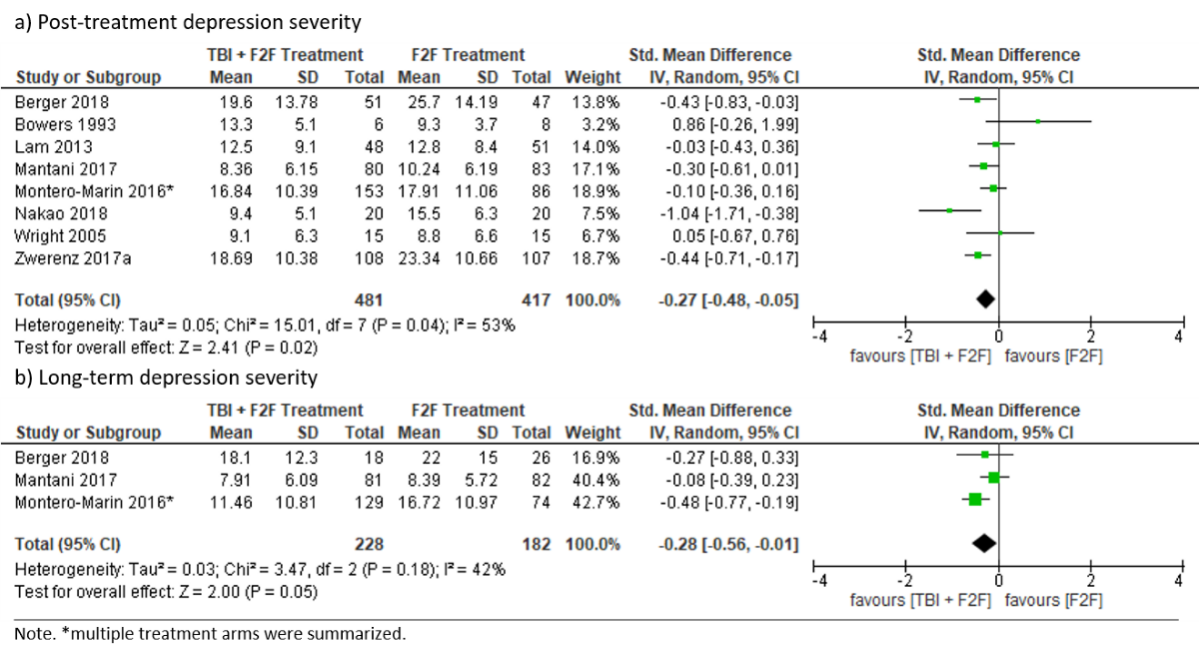
Forest plots for blended treatments (superiority trials).

Depression severity was significantly lower at posttreatment in blended treatment groups compared to f2f treatment controls, with substantial heterogeneity (SMD –0.27, 95% CI –0.48 to –0.05; *I*²=53%; 95% PI –0.88 to 0.34). Treatments did not differ significantly concerning long-term (4 months to 15 months) depression severity (SMD –0.28, 95% CI –0.56 to –0.01; *I*²=42%; 95% PI –3.13 to 2.57). There were no data available for dropouts concerning superiority trials (see Figure 10). Heterogeneity (*I*²=53%) for posttreatment depression severity may be explained by an outlying, small-sample study [69] favoring the blended treatment condition more clearly, which might have been due to the provision of a more intensive treatment regimen, since patients received blended treatment (internet-based TBI combined with f2f CBT) in addition to TAU consisting of f2f psychiatric care. Excluding this study resulted in decreased heterogeneity (SMD –0.22, 95% CI –0.40 to –0.03; *I*²=37%) and did not change the direction of effect.

### Collaborative Care Approach

**Figure 11:**
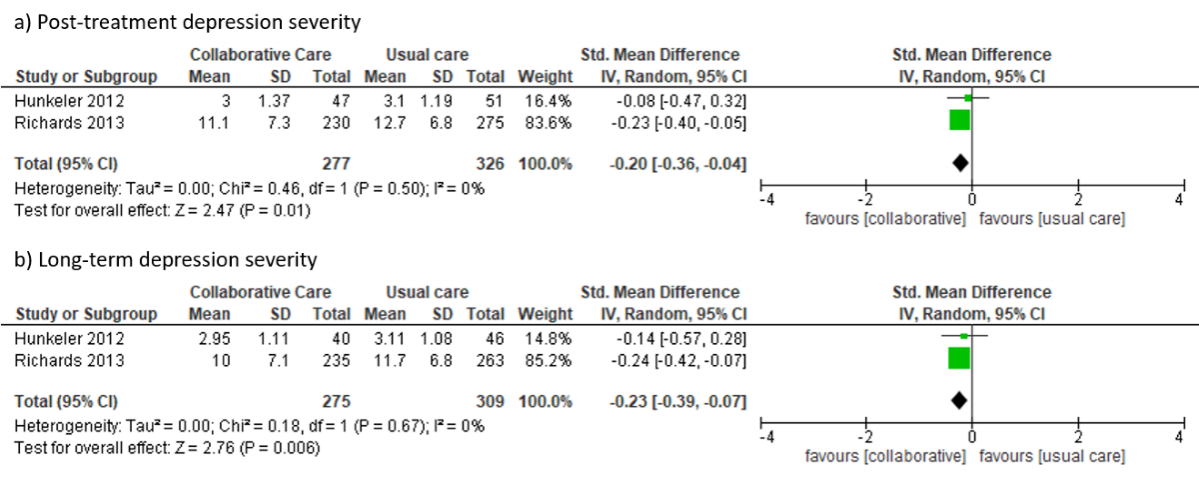
Forest plots for collaborative care approaches.

Three RCTs were identified applying TBIs, which were tested against usual care arms [43,79,80], in the context of a collaborative care approach. TBIs delivered in the context of collaborative care trials yielded lower posttreatment (SMD –0.20, 95% CI –0.36 to –0.04; *I*²=0%) and long-term (12 months: SMD –0.23, 95% CI –0.39 to –0.07; *I*²=0%) depression severity compared to usual care arms (see Figure 11).

### Stepped Care Approach

Two RCTs using TBIs in the context of a stepped care approach were identified in the course of the search update. The studies were too heterogeneous for evidence syntheses, since one study tested a stepped care approach (first step: internet-based treatment, second step: telephone-based treatment) against telephone-based treatment alone [67], and the other study tested an internet-based intervention against a waitlist control group as a first step within a stepped care approach [78].

## Discussion

### Principal Findings

Our study found that when compared to different control conditions, TBIs were more effective not only when delivered as stand-alone interventions but also when they were delivered as blended treatments or in collaborative care trials for people with diagnosed depression. Dropout rates did not differ between TBI and control conditions; however, assessment of TBI acceptance was limited due to underpowered comparisons. In addition, relevant statistical heterogeneity was a common finding for most meta-analytical comparisons. We included 78 RCTs comprising different application formats (stand-alone interventions [61/78, 78%], blended treatments [12/78, 15%], and stepped care [2/78, 3%] or collaborative care trials [3/78, 4%]), interventions, technologies for intervention delivery, clinical populations, and control groups.

### Stand-Alone Interventions

TBIs showed comparable effects to f2f treatments. Our findings are in line with a previous meta-analyses that found equivalent overall effects when comparing internet-based CBT to f2f treatment for mental disorders and somatic conditions on posttreatment symptom burden for studies on depressive symptoms specifically and for dropouts rates [100]. However, both results should be interpreted with caution, since both evidence syntheses were based on a limited number of studies.

When TBIs were tested against TAU controls, we found medium-to-small effects favoring TBIs concerning posttreatment and long-term depression severity. TAU was heterogeneous and consisted mostly of a mix of treatment options depending on the resources and routines of health care providers, general practitioner care, or care delivered in outpatient clinics. In addition, two-thirds of the studies included for this comparison also provided TAU in the TBI condition. Our results are in line with 2 previous meta-analyses that found a small effect favoring TBIs in comparison with TAU [101,102].

TBIs yielded beneficial medium-to-small effects on posttreatment and long-term depression severity when compared to attention placebo controls. To our knowledge, there is no previous meta-analysis available on this issue. However, the results are comparable to those comparing f2f psychotherapy with placebo [103] and pill placebo control groups [104].

We found a large effect in favor of the TBI group compared to waitlist controls for posttreatment and long-term depression severity. Our findings are in line with the only existing meta-analysis investigating TBIs in people with diagnosed depression [13]. This is not surprising, as there was a high overlap between the included studies. However, we were able to include more RCTs (+10) for the comparison of TBIs versus waitlist controls due to broader inclusion criteria and an updated literature search. Thus, our analysis emphasizes the robustness of the previous findings. However, the funnel plot on posttreatment depression severity was asymmetrical, with an emphasis on small studies depicting large differences in favor of TBIs compared to waitlist controls. Nevertheless, this is not a clear indicator of reporting bias because there are other sources (eg, heterogeneity, poor methodological quality) causing funnel plot asymmetry [16]. Between-study heterogeneity seems plausible to partly explain asymmetry, since we applied broad eligibility criteria and suspicious studies differed from the others in terms of population (postpartum depression) or publication year (1990), potentially resulting in more elevated differences.

Finally, TBIs did not result in lower posttreatment depression severity scores than no-treatment controls. This was not reasonable to expect, since no-treatment controls are comparable weak control groups, such as waitlist controls, which yield large effects when compared to TBIs [13]. Moreover, based on study reports, it cannot be ruled out that people allocated to the no-treatment control group made use of other health services for depression complaints (eg, care by a general practitioner), thus questioning whether true no-treatment controls were applied.

### Blended Treatments

We identified a small effect favoring blended treatments delivered in a superiority trial design compared to f2f treatments concerning posttreatment depression severity. Meta-analysis on blended treatments delivered in a noninferiority trial design (ie, substantial shortening of f2f contacts) did not reveal differences in posttreatment or long-term depression severity or on dropout rates compared to f2f treatments. To the best of our knowledge, there is no previous meta-analysis investigating the effectiveness and acceptance of blended treatments in people with depression. Additionally, despite extensive discussions on their potential usefulness for mental health care [105,106], there is no uniform definition of blended care/treatment as they are operationalized in different ways and rationales for blended treatments are often missing [105]. This was also the case in our study, since the concept of combining a TBI with an f2f treatment was usually explained insufficiently or not at all. In the included studies, it appears that blended treatments were implemented based on the motto more is more (intensification of the therapeutic dose by providing add-on treatment following a superiority trial design). Nevertheless, future studies could define and investigate more sophisticated variants of blended treatments, since there are many useful possibilities to enrich onsite therapy by, for example, fostering preprocessing and postprocessing of sessions or for diagnostic purposes in everyday life (eg, self-monitoring) [106].

### Collaborative Care Approach

TBIs delivered in the context of collaborative care yielded small effects on posttreatment and long-term depression severity when compared to TAU controls. However, findings should be viewed with caution, since only a few studies have been available until now, and investigated collaborative care approaches are heterogeneous. The identified posttreatment and long-term effects on depression severity are comparable to reported effects investigating collaborative care approaches without TBIs in comparison to usual care [107,108]. However, we do not know if and how much the technology-based component is involved in the effectiveness of these interventions, since collaborative care approaches are complex. Testing collaborative care approaches with and without a TBI component may help to determine the add-on benefit of this element and may be concurrently useful for a comparative cost-benefit analysis.

### Strengths and Limitations

Our review was conducted in line with Cochrane standards [16] and reported following PRISMA (Preferred Reporting Items for Systematic Reviews and Meta-Analyses) guidelines [109]. Additionally, studies were selected according to prespecified criteria [15]. We conducted a highly sensitive literature search considering key databases, databases of grey literature, and clinical trial registries without limiting the literature search to language. However, because of the extensive literature search, we deviated from the study protocol by omitting the forward and backward reference search. We structured and synthesized evidence using prespecified comparisons defined in the study protocol covering different application formats of TBIs in the acute treatment phase.

We applied broad inclusion criteria [15] contributing to observed heterogeneity regarding interventions, technologies for intervention delivery, psychotherapeutic rationales, and clinical populations in the included studies. Unfortunately, we were not able to explain statistical heterogeneity quantitatively (eg, by subgroup analyses) for most comparisons, since often only a few studies were available. However, we tried to explore heterogeneity narratively in these cases. In addition, when heterogeneity of the included studies is present (ie, *I*²≠ 0), the CI covers a narrower range than the PI of the respective comparison. Thus, pooled effects (SMDs) should be interpreted with caution: It may be that even if the pooled effect is significant (ie, CI not crossing null), the corresponding PI covers the null effect, meaning that in a new study conducted in a different setting (eg, different population), null treatment effects or effects in the other direction (harmful) may occur [20,110].

Although some information on dropouts [11] or treatment adherence [111] is addressed by most RCTs in this field, a comprehensive assessment of TBI acceptance was only partially possible, since data on dropouts were either missing or not usable (eg, data were only provided for one arm) or meta-analytic calculations were not possible (when no dropouts occurred in both study arms).

Considering the risk of bias ratings when interpreting the results, we found that the most common source of risk of bias was nonblinding of participants and personnel, followed by selective reporting and other bias. However, blinding of study participants is rarely possible in trials on TBIs.

### Conclusions

TBIs delivered as stand-alone interventions, blended treatments, or in collaborative care trials yield mostly beneficial effects in people with diagnosed depression. By investigating different application formats of TBIs, people being diagnosed with depression, and the long-term effectiveness of interventions, our results may be especially helpful to inform routine care. Given the potential transferability of our findings to routine care, we think that our findings may represent effectiveness (effectiveness under routine care), rather than efficacy (effectiveness under ideal conditions) of findings. Additionally, our results show a very consistent image of TBIs (it works), despite the clinical and methodological heterogeneity of the included studies.

However, there are still open questions that need to be addressed in future research. Even though dropouts are by far the most reported indicator for treatment acceptance/patient safety in studies with TBIs [11], data were often not usable for data synthesis resulting in underpowered comparisons for safety/acceptance assessment. Therefore, our findings with regard to this outcome should be interpreted with caution.

Additionally, safety assessments of TBIs considering different types of safety measures in people with diagnosed depression have not yet been conducted. Thus, to obtain a more comprehensive impression of the safety of TBIs, we suggest including all indicators according to Rozenthal et al [112] to evaluate negative events: (severe) adverse events, dropouts, nonresponse, novel symptoms, and unwanted events.

## Appendix

Multimedia Appendix 1 Summarizing table for meta-analysis.

Multimedia Appendix 2 Characteristics of included studies.

Multimedia Appendix 3 Therapeutic rationale for technology-based psychological interventions.

Multimedia Appendix 4 Risk of bias ratings (study level).

Multimedia Appendix 5 Funnel plots.

## Abbreviations

cCBT: computerized cognitive behavioral therapy
f2f: face-to-face
ITT: intention-to-treat
PI: prediction interval
PRISMA: Preferred Reporting Items for Systematic Reviews and Meta-Analyses
PROSPERO: International Prospective Register of Systematic Reviews
RCT: randomized controlled trial
RR: risk ratio
SMD: standardized mean difference
TAU: treatment as usual
TBI: technology-based psychological intervention
TIDECA: Comparative Effectiveness of Technology-Based Interventions in Different Steps of Depression Care
