# Supplementary material for: Effectiveness and Acceptance of Technology-Based Psychological Interventions for the Acute Treatment of Unipolar Depression: Systematic Review and Meta-analysis
Source: J Med Internet Res. 2021 Jun 13;23(6):e24584. doi: 10.2196/24584 (PMC8386371; doi:10.2196/24584)
Supplement: Multimedia Appendix 1 [file jmir_v23i6e24584_app1.pdf]

Appendix 1. Summary of results sorted by format of implementation of TBIs

	Outcomes		
**Comparisons**	**Post-treatment Depression severity**	**Follow-up Depression severity**	**Dropouts**
**TBIs as stand-alone interventions**			
TBI vs f2f treatment (n=6)	N.S.	N.S.	N.S.
TBI vs Treatment as usual (n=12)	Favours TBI group*	Favours TBI group	No data
TBI vs attention placebo (n=12)	Favours TBI group*	Favours TBI group	N.S.*
TBI vs waiting list (n=20)	Favours TBI group*	Favours TBI group*	N.S.
TBI vs no-treatment control (n=3)	N.S.*	No data	N.S.**
TBI vs TBI (n=21)	Not pooled	Not pooled	Not pooled
Other comparisons (n=2)	Not pooled	Not pooled	Not pooled
**TBIs within blended treatments**			
Non-inferiortity trials (n=3)	N.S.	N.S.	N.S.*
Superiority trials (n=8)	Favours TBI group*	N.S.	No data
**TBIs within collaborative care approaches**			
Collaborative care trials with TBIs vs usual care (n=3)	Favours TBI group	Favours TBI group	No data
**TBIs within stepped care approaches**			
TBI vs waiting list as a first step in a stepped care approach (n=1); Stepped care approach incorporating different TBIs vs TBI alone (n=1)	Not pooled	Not pooled	Not pooled

Note. N.S. = not statistically significant; TBI = technology-based psychological intervention;*substantial statistical heterogeneity ($I^2 \geq 50\%$);**results are based on data from one study.
