# Supplementary material for: Effectiveness and Acceptance of Technology-Based Psychological Interventions for the Acute Treatment of Unipolar Depression: Systematic Review and Meta-analysis
Source: J Med Internet Res. 2021 Jun 13;23(6):e24584. doi: 10.2196/24584 (PMC8386371; doi:10.2196/24584)
Supplement: Multimedia Appendix 2 [file jmir_v23i6e24584_app2.pdf]

Appendix 2. Characteristics of included studies (N = 78).

Study ID (Author / year / reference)	N	Study arms	Implementation in mental health care	Age – M (SD)	Sex (% of females)	Depression Diagnosis	Applied technologies	Interventions' duration (in weeks) and planned number of modules / sessions	Therapeutic rationale of TBIs
Agyapong (2017) [22]	73	1) Supportive text messages<br>2) control text messages	Stand-alone intervention	ND	68.5	MDD	Internet application on mobile phone	13,05 weeks / two text messages per day for 90 days	CBT
Andersson (2013) [23]	69	1) Internet-based cognitive behavioral therapy<br>2) in-person group cognitive behavioral therapy	Stand-alone intervention	42.3 (13.5)	78.3	MDD (71.0% in recurrent episode, 1.5% in single episode, Dysthymia (6.0%), MDD plus dysthymia 21.7%)	Internet-delivered text modules	8 weeks / 7 modules	CBT
Arjadi (2018) [24]	313	1) Internet-based behavioral activation intervention<br>2) Online psychoeducation	Stand-alone intervention	ND	80.8	MDD (mild (24.2%), moderate (31.2%), severe 40.90%, based on PHQ-9)	Online program (accessible via computer or mobile device) + telephone support	ND / 8 modules	BA
Berger (2011)* [25]	76	1) Guided internet-based CBT<br>2) unguided internet-based CBT<br>3) waiting list	Stand-alone intervention	38.8 (14.0)	69.7	MDD, Dysthymia (9.2%); MDD severity (mild (22.4%), moderate (60.5%), severe (7.9%), based on BDI-II scores)	1), 2) Online intervention	10 weeks / 11 modules	1), 2) CBT

Berger (2018) [26]	98	1) regular f2f psychotherapy plus internet-based CBT (Deprexis)<br>2) regular f2f psychotherapy	Blended treatment	43.1 (12.0)	66.3	(ICD-10; F32.- Depressive Episode, F33.- Recurrent Depressive Disorder, F34.- Persistent Affective Disorder, F38.- Other Affective Disorder, F39 Unspecified Affective Disorder)	Online program	12 weeks (length of Deprexis) / ND	CBT
Blackwell (2015) [27]	150	1) imagery cognitive bias modification<br>2) control condition (without training contingency, which was provided for other intervention arm)	Stand-alone intervention	ND	68.7	MDD	Online program (access via web browser)	4 weeks / 12 sessions	CBM
Bowers (1993) [28]	22	1) TAU<br>2) TAU plus therapist delivered CBT<br>3) TAU plus computer assisted CBT	Blended treatment	ND	68.2	MDD	3) Computer program	ND / 8 sessions	CBT
Carlbring (2013) [29]	80	1) Computer-administered therapy<br>2) waiting list	Stand-alone intervention	44.5 (13.5)	82.5	MDD	1) Online intervention, CD-ROM (mindfulness and acceptance instructions exercises)	8 weeks / 7 modules	1) BA with influences from acceptance and commitment therapy
Celano (2017) [30]	65	1) Positive psychology<br>2) cognition focused condition	Enhanced stand-alone intervention	ND	69.2	MDD	Telephone	6 weeks / 6 sessions	PP, CF (cognition focused emotionally neutral memory recall)
Choi (2012) [31]	63	1) Internet-based CBT (attuned to Chinese people	Stand-alone intervention	39.0 (11.7)	80	MDD	1) Online intervention, telephone (for	8 weeks / 6 online	1) CBT

		with depression living in Australia) 2) waiting list					supporting contacts)		
Choi (2014) [32]	158	1) Videoconferencing problem solving therapy, 2) face-to-face problem solving therapy 3) telephone support calls	Stand-alone intervention	64.8 (9.2)	78.5	MDD (65.2%), depressive disorder, NOS (29.7%), dysthymia (5.1%)	1) Laptop (Skype), 3) telephone	ND / 6 sessions	1) Problem solving therapy, 3) no specific rationale applied
Corruble (2016) [33]	221	1) Social rhythm therapy plus face-to-face treatment with a psychiatrist and antidepressant medication 2) clinical management plus face-to-face treatment with a psychiatrist and antidepressant medication 3) TAU (posteriori matched control group)	Blended treatment	ND	ND	MDD	1), 2) Telephone	1) 8 weeks / 8 sessions, 2) 8 weeks / ND	1) Social rhythm therapy, 2) Intensive clinical management (as described in the paper)
Dennis (2020) [35]	241	1)Telephone interpersonal psychotherapy + locally available standard postpartum care (including postpartum depression service), 2) locally available standard postpartum care	Enhanced stand-alone intervention	ND	100	MDD (postpartum)	1) Telephone-delivered	12 weeks / 12 modules	1) IPT

		(including postpartum depression service)							
Egede (2015) [36]	241	1) Videoconferencing behavioral activation treatment<br>2) face-to-face behavioral activation treatment	Stand-alone intervention	63.9 (5.1)	2	MDD	1) Videophone	8 weeks / 8 sessions	2) Behavioral activation
Flygare (2020) [37]	95	1) Internet-based CBT, 2) online psychoeducation control condition	Stand-alone intervention	45.30 (12.20)	75.80	MDD (71.6%), dysthymia (9.5%), Depressive Disorder NOS (4.2%), MDD in partial remission (15.8%)	1) Online intervention	8 weeks / 8 modules	1) CBT
Forand (2018) [38]	90	1) Internet-based CBT<br>2) waiting list	Stand-alone intervention	ND	75	MDD (98%)	1) Online Intervention, telephone (contacts with coaches)	8 weeks / 8 sessions	1) CBT
Forsell (2017) [39]	42	1) Internet-based CBT plus antenatal TAU<br>2) antenatal TAU	Enhanced stand-alone intervention	ND	100	MDD	Online platform	10 weeks / 10 modules	CBT
Gilbody (2015)* [40]	691	1) Internet-based CBT (Beating the Blues) plus usual GP care<br>2) internet-based CBT (MoodGYM) plus usual GP care)<br>3) usual GP care	Enhanced stand-alone intervention	39.9 (12.7)	67	MDD (81%)	1), 2) Online intervention, telephone (for technical support)	1) ND / 8 sessions, 2) ND / 5 sessions	1), 2)

Gili (2020) [41]	221	1) Online healthy lifestyle program, 2) online mindfulness program, 3) online positive affect promotion, 4) improved TAU	Enhanced stand-alone intervention	ND	77.8	MDD or dysthymia	1) & 2), Online intervention	4 to 8 weeks / 4 modules	1) Mindfulness, 2) Positive affect promot-ion
Graaf (2009)* [34]	303	1) Internet-based CBT plus TAU 2) internet-based CBT 3) TAU	1)Enhanced stand-alone intervention 2) stand-alone intervention	ND	56.8	Recurrent MDD (36.30%), single MDD (43.6%)	1), 2) Online intervention	1), 2) ND / 9 sessions	1), 2) CBT
Hunkeler (2012) [43]	103	1) Collaborative care website plus usual specialty mental health care 2) usual specialty mental health care	Collaborative care approach	ND	79.6	Recurrent MDD	1) Online platform	52 weeks / ND	CBT
Hur (2018) [44]	48	1) App-based cognitive restructuring, 2) App-based mood chart control group	Stand-alone intervention	ND	88.2	Other specified depressive disorder	1) Online intervention	3 weeks / ND	1) CT (especially cognitive restructuring)
Jannati (2020) [45]	78	1) App-based CBT, 2) waiting list	Stand-alone intervention	ND	100	MDD (postpartum)	1) Online intervention	8 weeks / 8 modules	1) CBT

Johansson (2012a) [48]	92	1) Internet-based psychodynamic treatment<br>2) structured support control group	Stand-alone intervention	45.6 (14.0)	75	Acute MDD (65.2%), partial remitted MDD (34.8%)	1) Online intervention	10 weeks / 9 modules	Psychodynamic therapy
Johansson (2012b)* [49]	121	1) Tailored internet-based CBT<br>2) standardized internet-based CBT<br>3) participation in online discussion group during waiting period	Stand-alone intervention	44.7 (12.1)	71.1	Acute MDD (73.6%), partial remitted MDD (26.4%), dysthymia (14.0%)	1), 2) Online Intervention	1) 10 weeks / 25 chapters, 2) 10 weeks / 8 chapters	1), 2) CBT
Johansson (2013) [47]	100	1) Internet-based psychodynamic treatment<br>2) waiting list plus continuous contact with therapist	Stand-alone intervention	ND	ND	Depression and anxiety disorders, data are reported separately for the depressed subgroup (MDD)	1) Online intervention<br>2) online contact to therapist	1) 10 weeks / 8 modules, 2) ND / ND	1) Psychodynamic therapy, 2) basic support from therapists
Johansson (2019) [46]	54	1) Internet-based CBT, 2) waiting list	Stand-alone intervention	ND	57.0	Recurrent MDD (94%)	1) Online intervention	8 weeks / 8 modules	1) CBT
Kessler (2009)[50]	297	1) Online CBT plus usual GP care<br>2) usual GP care while on waitlist	Enhanced stand-alone intervention	ND	68.0	MDD (mild (5.1%), moderate (26.2%), severe (68.7%), based on BDI score)	Online intervention (with a therapist online in real time)	16 weeks / 10 sessions	CBT

Kivi (2014) [51]	92	1) Internet-based CBT<br>2) TAU	Stand-alone intervention	36.6 (11.3)	66	MDD (mild (19%), moderate (34%), severe (41%), based on BDI-II cut-off scores)	Online program, CD (containing mindfulness and acceptance instructions), telephone (contact to coach)	12 weeks / 7 modules	CBT (consisting of BA, Acceptance and Commitment Therapy, and Mindfulness)
Kooistra (2019) [52]	103	1) Blended CBT, 2) f2f CBT	Blended treatment	ND	63.0	MDD	1) Online intervention	10 weeks / 10 f2f sessions and 9 web-based sessions	1) CBT
Hirsch* (2018) [42]	79	1) Online Cognitive Bias Modification with or, 2) without priming of repetitive negative thinking<br>3) Neutral task control condition	Stand-alone intervention	ND	78.5	GAD or MDD (outcome data was reported separately)	Online platform	3 weeks / 10 sessions	CBM
Lam (2013) [53]	105	1) Telephone CBT plus medication, 2) medication plus adherence reminder	Blended treatment	ND	54.6	Single MDD (33.33%), recurrent MDD (66.66%)	1) Telephone	8 to 10 weeks CBT; 12 weeks escitalopram / 8 sessions of CBT	CBT
Lang (2012) [54]	28	1) Imagery-focused cognitive Bias modification, 2) Control condition with same stimulus material, but with modified contingencies	Stand-alone intervention	ND	76.9	MDD	Computer program	1 weeks / 7 sessions	Imagery-focused CBM

Lappalainen (2015) [55]	39	1) Internet-based acceptance and commitment therapy<br>2) waiting list	Stand-alone intervention	51. 9 (12.9)	71.8	MDD (mild (25.6%), moderate (43.6%), severe (17.9%), based on BDI-II scores)	Online intervention	7 weeks / 6 modules	Acceptance and commitment therapy
Lindner (2014) [56]	38	1) Self-help program plus telephone support by therapist<br>2) Self-help program plus e-mail support by therapist	Stand-alone intervention	ND	84.2	MDD (84.2%), MDD + Dysthymia (15.8%)	1) Online program, telephone (contact to therapist), CD-ROM (with mindfulness and acceptance instructions)<br>2) Online program, e-mail (contact to therapist), CD-ROM (with mindfulness and acceptance instructions)	8 weeks / 7 modules	BA (with some influence from acceptance and commitment therapy)
Löbner (2018) [57]	647	1) Online CBT plus TAU<br>2) TAU	Enhanced stand-alone intervention	ND	68.5	MDD	Online program	ND / 5 modules	CBT
Luxton (2016) [58]	121	1) In Home treatment<br>2) in-person treatment	Stand-alone intervention	ND	18.2	MDD (9% suffered from minor depression)	1) Videoconferencing via laptop	8 weeks / 8 sessions	BA
Ly (2014) [60]	84	1) Smartphone-based BA intervention<br>2) smartphone-based mindfulness intervention	Stand-alone intervention	36.1 (10.8)	70	MDD, MDD and Dysthymia (49%)	1) Smartphone application<br>2) smartphone application	8 weeks / 3 chapters	1) BA, 2) Mindfulness practice programme

Ly (2015) [59]	93	1) Blended treatment (face-to- session plus smartphone application that were used between the sessions)<br>2) Face-to-face BA	Blended treatment	30.6 (11.4)	69.9	MDD	1) Smartphone application	1) 9 weeks / 4 face-to-face sessions (smartphone app was used between sessions),<br>2) 10 weeks / 10 sessions	1), 2) BA
Mantani (2017) [61]	164	1) Smartphone CBT plus medication change<br>2) medication change alone	Blended treatment	ND	53.1	MDD (with antidepressant resistance defined as scoring ≥ 10 on the BDI-II)	1) Smartphone application	9 weeks / 8 sessions	CBT
Meyer (2015) [62]	163	1) Internet-based CBT plus permission of using TAU, 2) TAU	Enhanced stand-alone intervention	42.0 (11.4)	74.8	MDD (37.2%), Dysthymia (10.4%), MDD and Dysthymia (39.3%), Neither MDD nor Dysthymia (12.9%)	1) Online intervention plus daily text messages (optionally)	13,05 weeks / ND	CBT
Milgrom (2016) [63]	43	1) Internet-based CBT<br>2) TAU	Stand-alone intervention	ND	100	Recurrent MDD (67.4%), single MDD (32.3%)	1) Online intervention	8 weeks / 6 sessions	CBT
Mohr (2011) [64]	85	1) Telephone CBT<br>2) TAU	Stand-alone intervention	55.9 (10.6)	9.4	MDD	1) Telephone	20 weeks / 16 sessions	CBT

Mohr (2012) [66]	325	1) Face-to-face CBT<br>2) Telephone CBT	Stand-alone intervention	ND	77.5	MDD	2) Telephone administered intervention	18 weeks / 18 sessions	CBT
Mohr* (2013) [65]	102	1) Guided internet-based CBT<br>2) unguided internet-based CBT<br>3) waiting list	Enhanced stand-alone intervention	ND	71.4	MDD	1) Online intervention, telephone (contact to coach)<br>2) online intervention	1), 2) 12 weeks / 18 lessons	1), 2) CBT
Mohr (2019) [67]	312	1) Stepped care program (1st step iCBT, 2nd step telephone CBT),<br>2) telephone CBT alone	Stepped care approach	ND	73.4	MDD	1) Online intervention, 2) telephone-delivered intervention	1) & 2), duration of the treatment depended on treatment success, but treatment lasted up to a maximum of 20 weeks	1) & 2), CBT
Montero-Marin* (2016) [68]	296	1) Self-guided online program plus improved TAU<br>2) therapist-guided online program plus improved TAU<br>3) improved TAU	Blended treatment	43.2 (9.3)	79.2	MDD	1) Online intervention<br>2) online intervention	1), 2): 13,05 weeks (estimated) / 10 module	1), 2) CBT
Nakao (2018) [69]	40	1) Blended CBT plus continuation with TAU with their treating psychiatrist<br>2) waiting list and continuation with TAU with their treating psychiatrist	Blended treatment	40.2 (9.7)	50	MDD	1) Online program	12 weeks / 5 core components	CBT

Nyström (2017)* [70]	312	1) Physical activity without rationale 2) physical activity with rationale, 3) BA based on Lewinsohn's model 4) BA based on Martell's model, 5) waiting list	Stand-alone intervention	42.0 (13.5)	76	MDD	3)-4) Online intervention	3)-4) 12 weeks / 8 sessions	3)-4) BA
Oehler (2020) [71]	348	1) Internet-based CBT, 2) progressive muscle relaxation control group	Stand-alone intervention	ND	78.7	Recurrent MDD (92.51%), single MDD (1.72%), dysthymia (23.92%)	1) Online intervention	6 weeks / 6 modules	1) CBT
O'Mahen (2014) [72]	83	1) BA 2) TAU	Stand-alone intervention	ND	100	MDD	1) Online treatment, telephone (support)	ND / 12 sessions	BA
Perini (2009) [73]	48	1) Online CBT 2) waiting list	Stand-alone intervention	49.3 (12.1)	77.8	MDD	1) Online intervention	8 weeks / 6 sessions	CBT
Pfeiffer (2020) [74]	330	1) Peer-supported cCBT, 2) enhanced usual care	Enhanced stand-alone intervention	51.6 (14.9)	20.0	MDD	1) Online intervention	13 weeks / 8 modules	1) CBT
Pihlaja (2020) [75]	100	1) iCBT plus add-on telephone support, 2) iCBT only	Stand-alone intervention	63.11 (11.10)	66.00	MDD	1) & 2), Online intervention	ND / 7 modules	1) & 2), CBT
Reins (2019) [76]	131	1) Internet-based CBT 2) online psychoeducation	Stand-alone intervention	41.6 (10.8)	75.6	MDD	1)Online intervention	ND / 6 sessions	CBT
Ren (2016) [77]	62	1) Internet-based CBT 2) waiting list	Stand-alone intervention	ND	ND	MDD	1) Online intervention	3 weeks / 5 sessions	CBT
Richards (2013) [79]	581	1) Collaborative care intervention plus TAU 2) TAU	Collaborative care approach	44.8 (13.3)	71.9	MDD (mild (14.3%), moderate (55.6%), severe (29.9%), based on ICD-10 diagnosis)	1) Telephone	14 weeks / 6 to 12 contacts	BA

Richards (2020) [78]	169	1) Immediate iCBT (step 2 within a stepped care approach), 2) waiting list	Stepped care approach	ND	ND	MDD	1) Online intervention	8 weeks / ND	1) CBT
Rollman [80] (2018)	704	1) Internet-based CBT supported by collaborative care manager<br>2) Internet-based CBT supported by collaborative care manager plus access to internet support group<br>3) TAU	Collaborative care approach	42.7 (14.3)	79.8	MDD (81.2%), generalized anxiety disorder (53.5%), panic disorder (15.8%), MDD and anxiety disorder (72.3%)	1) Online intervention, telephone (for care managers contact)<br>2) Online intervention, telephone (for care managers contact),	1), 2) 26.1 weeks / 8 sessions	1),2) CBT
Rosso (2017) [81]	77	1) Internet-based CBT<br>2) monitored attention control	Stand-alone intervention	ND	68.8	Recurrent MDD (74.0%), single MDD (26.0%)	1) Online intervention, telephone (for supporting contacts)	10 weeks / 6 lessons	CBT
Sandoval (2017) [82]	45	1) Interactive media-based, computer-delivered depression treatment program<br>2) no treatment control condition	Stand-alone intervention	ND	62.0	MDD (95%), dysthymia (5%)	1) Computer-delivered intervention (at study site)	6 weeks / 6 sessions	PST
Schuver (2016) [83]	40	1) Yoga intervention<br>2) walking control	Stand-alone intervention	42.7 (4.95)	100	MDD	DVD (for Yoga or walking, telephone (for contact to counsellors)	12 weeks / 8 sessions	Mindfulness-Bases Stress Reduction (MBSR)

Selmi (1990) [84]	36	1) Computer-administered therapy<br>2) therapist-administered therapy<br>3) waiting list	Stand-alone intervention	28.2 (5.0)	63.9	recurrent MDD (19.44%), single MDD (80.6)	1) Computer-delivered intervention (at study site)	6 weeks / 6 sessions	CBT
Smith (2017) [85]	270	1) Internet-based CBT, 2) self-help book (CBT), 3) self-help book (meditation), 4) waiting list	Stand-alone intervention	ND	81.9	MDD	1) Online intervention, optional email/telephone contact to technician/clinicia n	12 weeks / 6 modules	CBT
Steinmann (2020) [86]	59	1) Telephone CBT with reminding letters<br>2) telephone CBT without reminding letters	Stand-alone intervention	45.5 (13.6)	72	MDD	1) Telephone-delivered<br>2) telephone delivered	9 to 13 weeks / at least 9 sessions	CBT
Thase (2018) [87]	154	1) Blended CBT treatment<br>2) face-to-face CBT	Blended treatment	46.3 (14.3)	66.2	MDD	1) Online intervention	16 weeks / 9 modules (online) plus 12 face-to-face sessions	CBT
Titov (2010)* [88]	141	1) Internet-based CBT plus technician support<br>2) Internet-based CBT plus clinician support<br>3) waiting list	Stand-alone intervention	43.0 (12.9)	74	MDD	1), 2) Online intervention, telephone (for clinician/technicia n contact)	1), 2) 8 weeks / 6 lessons	1), 2) CBT

Titov (2011) [89]	77	1) Internet-based CBT<br>2) waiting list	Stand-alone intervention	43.9 (14.6)	73	MDD (51%) (results for depression subgroup reported separately)	1) Online intervention, contact to clinician via instant messaging or telephone	10 weeks / 8 modules	CBT
Torkan (2014) [90]	39	1) Positive imagery cognitive bias modification,<br>2) non-imagery control condition	Stand-alone intervention	ND	64.1	MDD	1), 2) Computer-delivered intervention	1 week / 7 sessions	1) CBM
Vernmark (2010)* [91]	88	1) Email therapy,<br>2) internet-based, CBT<br>3) waiting list	Stand-alone intervention	36.8 (12.9)	68.2	MDD	1), 2) Online Intervention	1), 2) 8 weeks / 8 modules	1), 2) CBT
Watkins (2012) [92]	121	1) Cognitive bias modification plus TAU<br>2) relaxation training plus TAU<br>3) TAU	Enhanced stand-alone intervention	ND	71.9	MDD	1)CD-ROM (with exercises), telephone	8 weeks / ND	CBM
Watts (2013) [93]	52	1) Mobile-based intervention (via an application)<br>2) computer-based intervention	Stand-alone intervention	41.0 (12.4)	80	MDD (mild (50%), moderate (23.5%), severe (14.7%), based on PHQ-9 severity Status)	1) Access to intervention via app (mobile phone or iPad)<br>2) Access to intervention via computer	1), 2) 8 weeks / 6 sessions	1), 2) CBT

Welch (2019) [94]	14	1) eCBT plus transcranial direct current stimulation, 2) eCBT plus sham stimulation	Stand-alone intervention	53.21 (12.48)	85.71	MDD	1) & 2), Computer-delivered intervention (at study site)	1) & 2), 4 weeks / ND	1) & 2), CBT
Williams (2013) [95]	69	1) imagery-focused cognitive bias modification plus internet-based CBT<br>2) waiting list	Stand-alone intervention	ND	76.2	MDD	1) Online interventions (CBM, CBT)	11 weeks / 7 CBM sessions plus 6 CBT sessions	1) CBM, CBT
Williams (2015) [96]	121	1) Positive imagery cognitive bias modification plus internet-based CBT<br>2) control cognitive bias modification condition plus internet-based CBT	Stand-alone intervention	ND	73.3	MDD	1), 2) Online interventions (CBM, CBT)	1), 2) 11 weeks / 7 active/control CBM sessions plus 6 CBT sessions	1) CBM plus CBT, 2) control CBM plus CBT

Wright (2005) [97]	45	1) Blended cognitive therapy 2) face-to-face cognitive therapy, 3) waiting list	Blended treatment	ND	75.6	single MDD	1) Computer-delivered intervention (at study site)	8 weeks / 9 face-to-face session followed immediately by 8 computer sessions	1) Cognitive therapy (or CBT)
Zagorscak (2018) [98]	108 9	1) Internet-based CBT plus standardized, automated feedback and contact on demand 2) internet-based CBT plus semi-standardized e-mail feedback	Stand-alone intervention	45.7 (11.3)	65.6	Single MDD (42.1%), remitted MDD (26.2%), dysthymia (8.3%), double depression (5.3%), Bipolar or NOS (6.0%), no current/past affective disorder (12.2%)	1), 2) Online intervention	6 weeks / 7 modules	1) ,2) CBT
Zwerenz [99] (2017)	611	1) Blended treatment (inpatient face-to-face sessions plus internet-based CBT), 2) Active control group (inpatient face-to-face sessions plus online information on depression)	Blended treatment	48.0 (9.8)	60.7	Recurrent MDD (65.9%), single MDD (31.9), depression severity: mild (0.01%), moderate (79.9%), severe (16.6%) according to ICD-10 classification.	1) Online intervention	12 weeks / 12 modules	1) CBT

Note. * = Study arms were summarized for meta-analysis. N = Number of participants randomized; M = Mean; SD = Standard deviation; MDD = Major depressive disorder; ND = No data; PHQ-9 = Patient Health Questionnaire 9 item; BDI = Beck Depression Inventory; CBT = Cognitive behavioral therapy; iCBT = Internet-based cognitive behavioral therapy; TAU = treatment as usual; NOS = Not otherwise specified; GP = General practitioner; GAD = Generalized anxiety disorder; BA = behavioral activation; f2f = face-to-face; CBM = Cognitive bias modification; TAU = Treatment as usual; PP = Positive psychology; CF = Cognition focused; CT = Cognitive therapy; PA = physical activity; PST = Problem solving therapy.
