# Supplementary material for: Effectiveness and Acceptance of Technology-Based Psychological Interventions for the Acute Treatment of Unipolar Depression: Systematic Review and Meta-analysis
Source: J Med Internet Res. 2021 Jun 13;23(6):e24584. doi: 10.2196/24584 (PMC8386371; doi:10.2196/24584)
Supplement: Multimedia Appendix 3 [file jmir_v23i6e24584_app3.pdf]

Appendix 3. Therapeutic rationales of TBI arms (n=101) in included studies.

**Therapeutic rationale of TBIs**	**Number of identified TBI-arms**	**Proportion (in %) on all TBI-arms***
Cognitive behavioral treatment approaches (behavioral activation [n=12], cognitive behavioral therapy [n=56], cognitive therapy [n=1])	83	82
Cognitive bias modification	7	7
Mindfulness	3	3
Problem solving therapy	2	2
Psychodynamic therapy	3	3
Acceptance and commitment therapy	1	1
Cognition focused treatment (ie, emotionally neutral memory recall)	1	1
Intensive clinical management	1	1
Positive affect promotion	1	1
Positive psychology	1	1
Social rhythm therapy	1	1

Note. *Overall percentage exceeds 100%, since three trials combined rationales (eg, psychodynamic therapy and mindfulness), and were thus considered repeatedly.
