# Supplementary material for: Effectiveness and Acceptance of Technology-Based Psychological Interventions for the Acute Treatment of Unipolar Depression: Systematic Review and Meta-analysis
Source: J Med Internet Res. 2021 Jun 13;23(6):e24584. doi: 10.2196/24584 (PMC8386371; doi:10.2196/24584)
Supplement: Multimedia Appendix 4 [file jmir_v23i6e24584_app4.pdf]

Appendix 4. Risk of bias ratings of included studies.

Studies	Random sequence generation (selection bias)	Allocation concealment (selection bias)	Blinding of participants and personnel (performance bias)	Blinding of outcome assessment (detection bias)	Incomplete outcome data (attrition bias)	Selective reporting (reporting bias)	Other bias
Agyapong 2017 [22]	+	+	-	+	+	?	+
Andersson 2013 [23]	+	+	-	?	+	?	-
Arjadi 2018 [24]	+	+	+	-	?	+	+
Berger 2011 [25]	+	+	-	+	+	?	-
Berger 2018 [26]	+	+	-	+	?	-	-
Blackwell 2015 [27]	+	+	+	+	+	-	+
Bowers 1993 [28]	?	?	-	+	-	?	-
Carlbring 2013 [29]	+	+	-	+	+	?	-
Celano 2017 [30]	+	+	-	+	+	+	+
Choi 2012 [31]	+	+	-	?	+	+	+
Choi 2014 [32]	+	+	-	-	+	?	-
Corruble 2016 [33]	+	+	-	?	?	?	?
Dennis 2020 [35]	+	+	-	+	+	?	-
Egede 2015 [36]	+	+	-	+	+	+	+
Flygare 2020 [37]	+	+	-	+	?	?	+
Forand 2018 [38]	+	+	-	-	-	-	-
Forsell 2017 [39]	+	+	-	-	+	-	-
Gilbody 2015 [40]	+	+	-	+	+	+	?
Gili 2020 [41]	+	+	-	+	-	-	-
Graaf 2009 [34]	+	+	-	+	+	-	-
Hirsch 2018 [42]	+	+	?	+	-	+	+
Hunkeler 2012 [43]	+	+	-	+	+	?	+
Hur 2018 [44]	+	+	-	+	-	?	+
Jannati 2020 [45]	+	+	-	+	+	+	?
Johansson 2012a [48]	+	+	-	-	+	-	+
Johansson 2012b [49]	+	+	-	+	+	-	+
Johansson 2013 [47]	+	+	-	?	+	?	+
Johansson 2019 [46]	+	+	-	+	?	?	?
Kessler 2009 [50]	+	+	-	+	+	?	-
Kivi 2014 [51]	+	+	-	+	-	?	-
Kooistra 2019 [52]	+	+	-	+	+	+	+
Lam 2013 [53]	+	+	-	+	+	-	-
Lang 2012 [54]	+	?	?	-	?	?	+
Lappalainen 2015 [55]	+	+	-	+	+	?	+
Lindner 2014 [56]	+	?	-	+	+	?	?
Löbner 2018 [57]	+	+	-	+	?	?	-
Luxton 2016 [58]	+	?	-	+	+	+	+
Ly 2014 [60]	+	+	-	+	+	-	+

Ly 2015 [59]	+	+	-	+	+	-	-
Mantani 2017 [61]	+	+	-	+	+	-	+
Meyer 2015 [62]	+	+	-	+	+	?	-
Milgrom 2016 [63]	+	+	-	+	+	?	+
Mohr 2011 [64]	?	?	-	+	+	+	+
Mohr 2012 [65]	+	+	-	+	+	-	+
Mohr 2013 [66]	+	+	-	+	+	?	+
Mohr 2019 [67]	+	+	-	+	+	?	?
Montero-Marin 2016 [68]	+	+	-	+	+	+	+
Nakao 2018 [69]	+	+	-	+	+	+	?
Nyström 2017 [70]	+	+	-	+	?	-	+
Oehler 2020 [71]	+	+	-	+	+	+	-
O´Mahen 2014 [72]	+	+	-	+	+	?	+
Perini 2009 [73]	+	?	-	+	+	-	+
Pfeiffer 2020 [74]	+	+	-	+	+	+	+
Pihlaja 2020 [75]	?	?	-	+	+	?	?
Reins 2019 [76]	+	+	-	?	+	-	-
Ren 2016 [77]	+	?	-	+	+	?	?
Richards 2013 [79]	+	+	-	+	+	-	-
Richards 2020 [78]	+	+	-	?	+	-	-
Rollman 2018 [80]	+	+	-	+	+	-	+
Rosso 2017 [81]	+	+	-	+	+	-	-
Sandoval 2017 [82]	+	?	-	+	+	?	?
Schuver 2016 [83]	+	?	-	+	-	-	+
Selmi 1990 [84]	?	?	-	+	+	?	-
Smith 2017 [85]	+	+	-	-	+	-	?
Steinmann 2020 [86]	+	+	-	+	+	-	+
Thase 2018 [87]	+	+	-	+	+	?	?
Titov 2010 [88]	+	?	-	?	+	-	+
Titov 2011 [89]	+	-	-	-	+	-	+
Torkan 2014 [90]	?	?	-	+	-	?	+
Vernmark 2010 [91]	+	+	-	+	+	?	+
Watkins 2012 [92]	+	+	-	+	+	-	+
Watts 2013 [93]	+	+	-	+	-	?	+
Welch 2019 [94]	?	?	+	+	?	?	+
Williams 2013 [95]	+	+	-	+	+	?	+
Williams 2015 [96]	+	+	+	+	+	?	+
Wright 2005 [97]	?	?	-	+	?	?	?
Zagorscak 2018 [98]	+	+	-	+	+	?	+
Zwerenz 2017 [99]	+	+	-	+	+	+	?

+	low risk
?	unclear risk
-	high risk
