# Supplementary material for: Effectiveness and Acceptance of Technology-Based Psychological Interventions for the Acute Treatment of Unipolar Depression: Systematic Review and Meta-analysis
Source: J Med Internet Res. 2021 Jun 13;23(6):e24584. doi: 10.2196/24584 (PMC8386371; doi:10.2196/24584)

Appendix 5. Funnel plots for different comparisons for post-treatment depression severity.

a) TBI. vs. treatment as usual

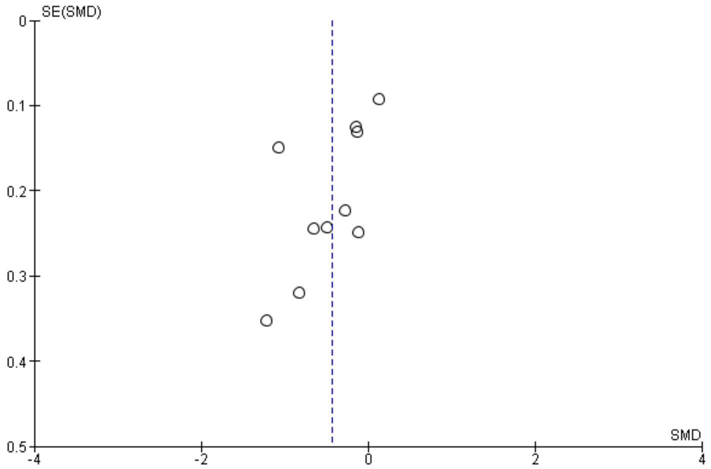

b) TBI vs. attention placebo

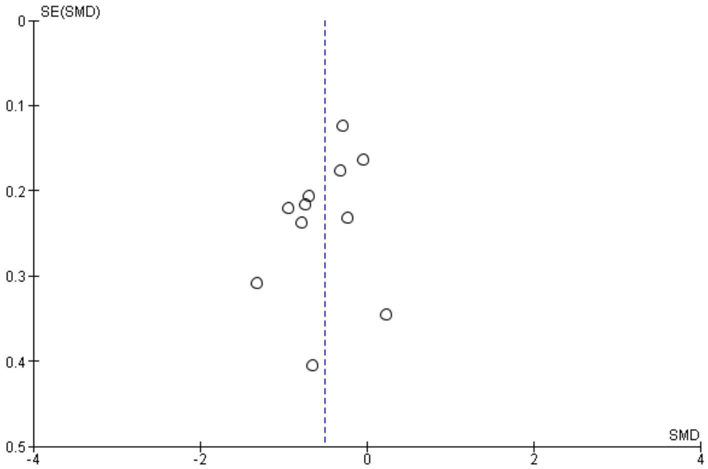

c) TBI vs. waiting list

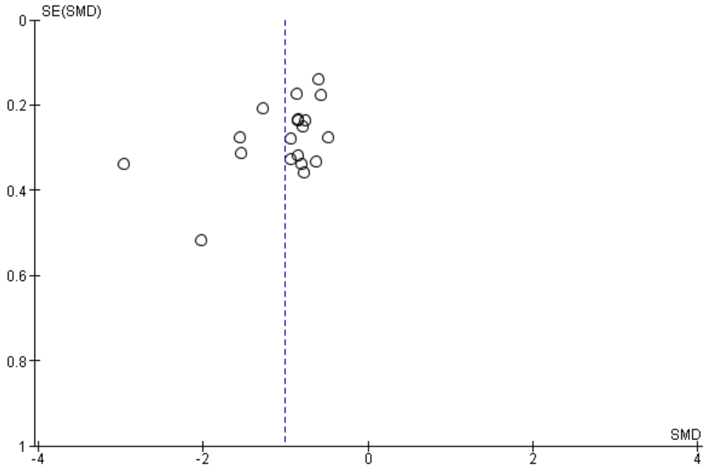

Supplement: Multimedia Appendix 5 [file jmir_v23i6e24584_app5.pdf]
